# Body fat measurement in adolescent girls with type 1 diabetes: a comparison of skinfold equations against dual‐energy X‐ray absorptiometry

**DOI:** 10.1111/apa.13366

**Published:** 2016-04-06

**Authors:** S Särnblad, A Magnuson, U Ekelund, J Åman

**Affiliations:** ^1^ Faculty of Medical SciencesSchool of MedicineÖrebro UniversityÖrebroSweden; ^2^ Faculty of Medical SciencesClinical Epidemiology and BiostatisticsÖrebro UniversityÖrebroSweden; ^3^ Department of Sport MedicineNorwegian School of Sport SciencesOsloNorway; ^4^ Medical Research Council Epidemiology UnitCambridgeUK

**Keywords:** Adolescents, Body composition, Dual‐energy X‐ray absorptiometry, Skinfold measurements, Type 1 diabetes

## Abstract

**Aim:**

Skinfold measurement is an inexpensive and widely used technique for assessing the percentage of body fat (%BF). This study assessed the accuracy of prediction equations for %BF based on skinfold measurements compared to dual‐energy X‐ray absorptiometry (DXA) in girls with type 1 diabetes and healthy age‐matched controls.

**Methods:**

We included 49 healthy girls and 44 girls with diabetes aged 12–19 years old, comparing the predicted %BF based on skinfold measurements and the %BF values obtained by a Lunar DPX‐L scanner. The agreement between the methods was assessed using an Bland–Altman plot.

**Results:**

The skinfold measurements were significantly higher in girls with diabetes (p = 0.003) despite a nonsignificant difference in total %BF (p = 0.1). A significant association between bias and %BF was found for all tested equations in the Bland–Altman plots. Regression analysis showed that the association between skinfold measurements and %BF measured by DXA differed significantly (p = 0.039) between the girls with diabetes and the healthy controls.

**Conclusion:**

The accuracy of skinfold thickness equations for assessment of %BF in adolescent girls with diabetes is poor in comparison with DXA measurements as criterion. Our findings highlight the need for the development of new prediction equations for girls with type 1 diabetes.

**Key Notes**

- Skinfold measurement is an inexpensive and widely used technique for assessing the percentage body fat.
- This study showed that using skinfold thickness equations to assess the percentage body fat in 44 adolescent girls aged 12–19 with type 1 diabetes was less accurate compared to measurements obtained using dual‐energy X‐ray absorptiometry as criterion.
- There is a need to develop new prediction equations for girls with type 1 diabetes**.**

## Introduction

There have been several reports of increased body mass index (BMI) in adolescents with type 1 diabetes in comparison with healthy controls. This difference has mainly been observed in girls 1, 2, 3, but some studies have reported similar differences in boys 4, 5, 6. The inference of these findings is that increased BMI reflects excessive fat accumulation, and this has been confirmed when body composition has been measured with dual‐energy X‐ray absorptiometry (DXA) 7 and skinfold thickness measurements 4, 8.

Skinfold measurements are noninvasive and inexpensive and have therefore been frequently used in studies of children and adolescents with type 1 diabetes 3, 4, 7, 8, 9. Multiple equations have been developed to predict the percentage of body fat (%BF) in healthy adolescents and young adults from skinfold measurements 10, 11, 12, 13, 14, 15, but none have been developed from skinfold measurements of adolescents with type 1 diabetes. To our knowledge, no study has validated the existing equations in a population of adolescents with type 1 diabetes.

The aim of this study was to validate the most commonly used skinfold equations to estimate %BF using body composition measurements by DXA as the criterion in adolescent girls with type 1 diabetes and to compare the associations with that of age‐matched healthy controls.

## Patients and methods

### Subjects

Data for 44 girls with type 1 diabetes and 49 healthy girls matched for age were pooled from two different studies conducted at the Department of Pediatrics, Örebro University Hospital, Sweden 7, 16. All the subjects and their parents gave informed consent, and the study was approved by the Ethics Committee of Örebro County Council.

### Body composition assessments

All measurements were performed in the fasting state in the morning before breakfast to minimise differences in hydration. Height and weight were measured, and BMI (kg/m^2^) was calculated for each subject. Weight was measured in light clothing to the nearest 0.1 kg, and height was measured to the nearest 0.5 cm. Waist circumference was measured at the level of the umbilicus.

Skinfold thickness was measured with a Harpenden calliper (British Indicators Ltd, West Sussex, UK) at the biceps, triceps, subscapular and suprailiac areas 17. Three skinfold measurements were performed at each site, and the mean of the three measurements was calculated. Two highly experienced investigators performed all the measurements.

Six skinfold equations were selected for validation, and these were derived from an original population with appropriate age and based on biceps, triceps, suprailiac and/or subscapular skinfolds 10, 11, 12, 13, 14, 15 (Table 1). The equation devised by Siri was used to convert body density to %BF using the equation %BF = 495/body density minus 450. Body composition was also measured using a Lunar DPX‐L scanner (Lunar Corp, Wisconsin, USA). The measurement gave a coefficient of variation (CV) for fat measurements of 10.4%, 1.7% and 0.3%, assessed in three different phantoms with a fat content of 10, 20 and 40 kg, respectively.

**Table 1:** Skinfold equations to estimate percentage body fat used in the study

Author	Number	Sex	Age	BF%	Criterion	Prediction equation
Slaughter et al. 10	136	F	8–29	Appr. 27.0	MC	BF% = 1.33*A −0.013*A^2^ −2.5 or when A > 35 mm BF% = 0.546*A + 9.7
Durnin and Rahaman 11	38 45	F	13.2–16.4 18.0–29.1	24.0 (4.9) 24.2 (6.5)	UWW	BD = 1.1369 −0.0598*logB BD = 1.1581 −0.072*log B
Deurenberg et al. 12	34	F	16.8	21.7	UWW	BD = 1.1830 −0.0813*logB
Sloan et al. 13	50	F	20.2 ± 1.7	22.9 (5.58)	UWW	BD = 1.0764 −0.00081 suprailiac −0.00088 triceps
Thorland et al. 14	133	F	16.5 ± 1.4	14.5 ± 4.3	UWW	BD = 1.0987 −0.00122C + 0.00000263C^2^
Parizkova et al. 15	62	F	13–16	Appr. 4–38	UWW	BD = 1.114 −0.031log triceps −0.041log subscapular

BF% = percentage body fat. BD = body density. A = triceps + subscapular skinfold (mm), B = triceps + biceps + subscapular + suprailiac skinfolds (mm), C = triceps + subscapular + suprailiac skinfolds (mm). MC = multicompartment model, UWW = underwater weighing.

### Laboratory measurements

Haemoglobin A1c (HbA1c) was measured by high‐pressure liquid chromatography using the Mono‐S standard 18. The values were converted to the International Federation of Clinical Chemistry and Laboratory Medicine (IFCC) standard using the equation IFCC (mmol/mol) = 10.45 multiplied by Mono−S(%) minus 10.62. The reference level for healthy subjects is 27–42 mmol/mol with the IFCC standard 19.

### Statistical analysis

Descriptive statistics were calculated using means, standard deviations (SD) and ranges. The unpaired t‐test was used to evaluate differences in the clinical characteristics variables between healthy controls and girls with type 1 diabetes. Agreement between %BF from DXA and estimated %BF from skinfold equations was assessed using the Bland–Altman methods 20.

Regression analysis was used to estimate the association between the sum of the triceps, biceps, suprailiac and subscapular skinfolds in millimetres and %BF from DXA. The two lines in Figure 2 are estimated from the nonlinear regression with %BF from DXA as the outcome variable. The sum of the skinfold measurements – linear and quadratic and group, namely type 1 diabetes or control patient – was used as independent variables in the regression.

A stepwise multiple regression analysis was used to calculate a prediction equation of %BF from skinfold values in girls with diabetes. %BF obtained by DXA was used as the dependent variable. Seven variables were included in the first model: BMI, age, log suprailiac skinfold, log biceps skinfold, log triceps skinfold, log subscapular skinfold and HbA1c. When we used a cut‐off level of p < 0.01, the final model included all the variables but age, log suprailiac and HbA1c. Stata Statistical Software release 9 (StataCorp, College Station, Texas, USA) was used for all statistical calculations.

## Results

### Clinical characteristics

Table 2 describes the clinical characteristics and shows that no significant differences were seen between the groups in age, height, weight, BMI or %BF. Triceps, subscapular and suprailiac skinfolds were significantly higher in the girls with diabetes than the controls.

**Table 2:** Clinical characteristics

	Controls (n = 49)			Type 1 diabetes (n = 44)			
	Mean	SD	Range	Mean	SD	Range	p‐value*
Age (years)	16.8	1.7	12.3–19.9	16.4	1.9	12.1–19.0	0.210
Weight (kg)	64.3	11.9	44.2–87.6	66.7	11.0	42.0–88.9	0.305
Height (m)	1.66	0.06	1.54–1.82	1.65	0.07	1.49–1.79	0.236
BMI (kg/m^2^)	23.1	3.7	17.4–31.1	24.5	3.3	16.5–31.1	0.062
Biceps skinfold (mm)	12.4	6.3	4.9–27.8	14.8	6.8	5.2–31.9	0.084
Triceps skinfold (mm)	20.6	7.1	9.2–34.1	24.3	7.0	8.9–36.8	0.014
Subscapular skinfold (mm)	16.3	7.7	6.6–35.4	21.1	11.1	5.3–54.1	0.016
Suprailiac skinfold (mm)	17.0	7.7	4.8–37.7	23.0	8.6	6.1–40.0	<0.001
Sum skinfolds (mm)	66.3	25.6	26.5–125.0	83.1	28.1	29.2–148.7	0.003
% body fat (DXA)	32.2	8.3	13.0–46.7	34.9	7.6	13.5–48.5	0.104
Waist circumference (cm)	76.6	9.2	62.0–97.5	79.2	9.4	61.0–100.0	0.173
HbA_1C_ (mmol/mol)				70.1	13.2	46.9–102.2	
Daily dosages of insulin (U/kg/d)				1.1	0.3	0.6–2.1	

*p‐values from *t*‐test.

### Comparison between estimated %BF by skinfold measurements and by DXA

Table 3 shows the results in terms of bias defined as observed BF% from DXA minus estimated %BF from the skinfold equations. All skinfold equations showed significantly statistically lower %BF among girls with type 1 diabetes in comparison with DXA, except the equations by Slaughter et al. 10 and Parizkova et al. 15. The findings in the healthy control group were similar, with significant underestimations of %BF by skinfold measurements in all equations, except for the equation by Parizkova et al. 15.

**Table 3:** Bias and 95% limits of agreement for percentage body fat predicted by skinfold thickness equations against DXA measurements

Equation	Control girls			Type 1 diabetes		
	Bias (95% CI)	95% limits of agreement	Corr (r)	Bias (95% CI)	95% limits of agreement	Corr (r)
Slaughter	2.9 (1.7–4.1)	−5.5 to 11.2	0.07^NS^	0.8 (−0.6 to 2.2)	−8.6 to 10	−0.4^S^
Durnin and Rahaman	1.4 (0.1–2.7)	−7.6 to 10.4	0.74^S^	1.1 (0.0–2.3)	−6.3 to 8.6	0.74^S^
Deurenberg	5.0 (3.9–6.2)	−2.9 to 12.9	0.51^S^	3.9 (2.9–4.9)	−2.4 to 10.2	0.45^S^
Sloan	8.2 (6.8–9.6)	−1.6 to 18.1	0.63^S^	7.3 (5.9–8.6)	−1.7 to 16.2	0.50^S^
Thorland	6.8 (5.7–8.0)	−1.4 to 15.1	−0.05^NS^	3.7 (2.3–5.1)	−5.3 to 12.7	−0.35^S^
Parizkova	−0.2 (−1.4 to 1.0)	−8.5 to 8.1	0.64^S^	−0.5 (−1.6 to 0.5)	−7.3 to 6.2	0.43^S^

Bias: Percentage body fat by dual‐energy X‐ray absorptiometry minus values from skinfold thickness equations. 95% limits of agreement: ± 2 SD of the mean difference between methods. r = correlation between bias and percentage body fat. S = significant, NS = nonsignificant.

**Figure 1:**
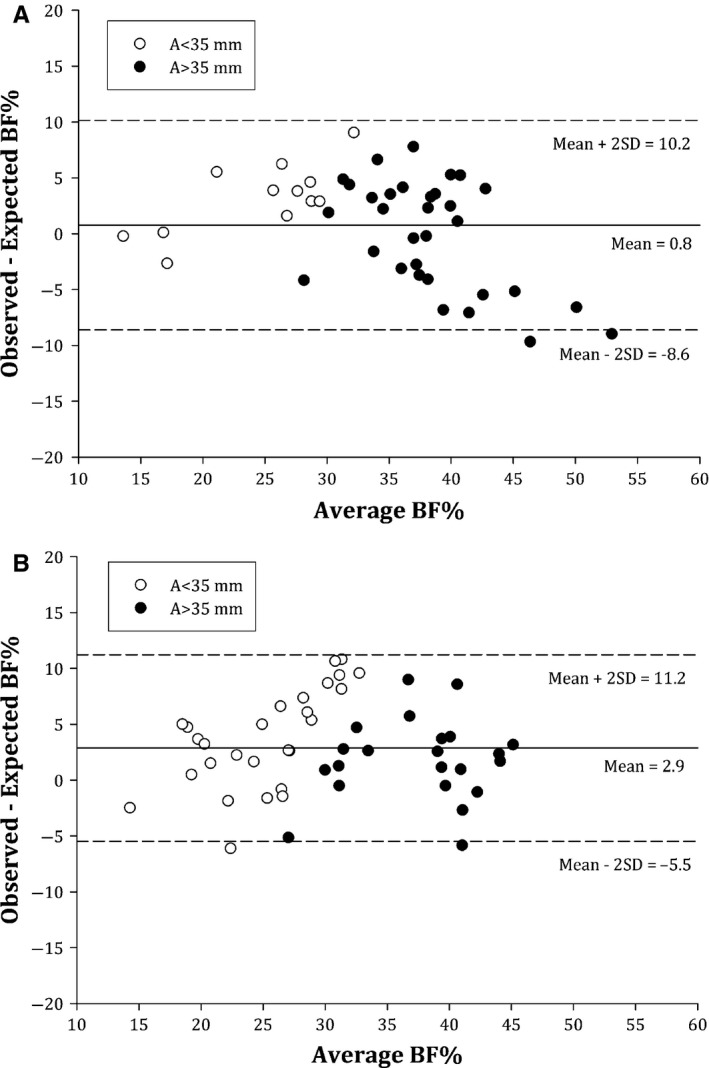
Comparison of predicted percentage body fat between skinfold equation by Slaughter et al. and measurements by DXA in girls with type 1 diabetes (A) and controls (B). Mean differences ± 2 SD for the difference are given in the Figure. White dots indicate when the sum of triceps and subscapular skinfold was less then 35 mm and black dots when the sum was more than 35 mm. Observed = %BF by dual‐energy X‐ray absorptiometry. Expected= %BF from skinfold thickness equation.

Bias was significantly correlated to the level of average %BF – the sum of the DXA and skinfold measurements divided by two − in all equations among girls with diabetes (Fig. 1). In four of the six equations, the correlation was positive, indicating higher bias and a possible underestimation of BF% by skinfold when the level of the average %BF was high. In the healthy control group, only two of the six equations, Slaughter 10 and Thorland 14, showed nonsignificant correlations.

### Relationship between the sum of the skinfold measurements and the %BF

**Figure 2:**
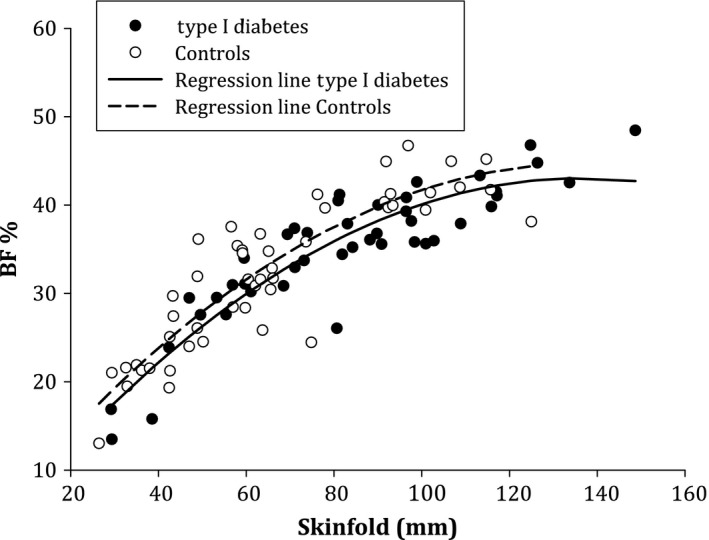
The relation between sum of skinfolds in millimetre and percentage body fat measured by DXA.

Regression analysis showed that the association between skinfold measurements and %BF measured by DXA differed significantly between the girls with diabetes and the healthy controls (Fig. 2)**.** For a given sum of skinfold, the control group had 1.6%‐units higher %BF measured by DXA (95% confidence interval 0.1–3.2, p = 0.039). As shown in Figure 2, the relationship between the sum of the skinfold measurements and %BF demonstrated a linear association for %BF of less than 35, whereas a levelling‐off effect was observed in individuals with higher %BF.

### Prediction equation of %BF in girls with type 1 diabetes

The following prediction equation for %BF was developed:

%BF = −20.284 + 10.715 × log biceps + 8.871 × log triceps + 6.856 × log subscapular + 0.9128 × BMI. This model explained 91% of the variance in %BF from DXA measurements with an adjusted r^2^ of 0.91.

## Discussion

The results from the present study suggest that all the prediction equations based on skinfold measurements that we evaluated, except those devised by Parizkova et al. 15 and Slaughter et al. 10, underestimated BF% in comparison with DXA in adolescent girls with type 1 diabetes. Furthermore, we observed a systematic bias for all tested equations, indicating that the prediction of percentage body fat from skinfold measurements deteriorates with increasing fatness.

One of the main findings in this study was that skinfold equations often underestimated %BF. Our results suggest that the sum of the skinfold measurements was significantly higher in girls with diabetes than healthy control girls, despite nonsignificant differences in BMI and %BF. This indicates a different relationship between skinfold measurements and total body fatness between the two groups. We have previously observed this phenomenon in middle‐aged diabetic patients with a long disease duration 21, and Tillman et al. 22 observed that girls and boys with diabetes had significantly thicker triceps and biceps skinfold than healthy adolescents, despite having a similar BMI.

One possible explanation for this could be increased stiffness in subcutaneous fat caused by glycated collagen 23. Skin collagen glycation has been associated with HbA1c and proposed as a predictor of microvascular complications 24. However, in our study of young girls with type 1 diabetes, very few other signs of diabetic complications were observed and we found no significant influence of Hba1c in our prediction equation. Therefore, it is possible that the increased subcutaneous stiffness was an early consequence of type 1 diabetes, preceding other types of long‐term effects. It is, however, also possible that there was a real difference in subcutaneous fat deposition between the groups, where girls with diabetes accumulated relatively more fat subcutaneously than healthy girls.

To our knowledge, there have been no previous validation studies in adolescents with type 1 diabetes, but cross‐validation studies in healthy adolescents have suggested that the equation by Slaughter et al. is valid for predicting %BF in girls 25, 26. Our observations in the healthy control group agreed with these findings. The mean bias was low (2.9%), and no systematic error was observed. This is comparable with previous cross‐validations in adolescent girls using underwater weighing (UWW) (bias 2%; limits of agreement ±13%) 27, DXA (bias 1.64%; limits of agreement ±7.4%) 26 or a four‐compartment model (bias 0.1%; limits of agreement ±10.2%) 25 as the criterion methods.

Skinfold measurements are often used in large‐scale studies to assess body composition. This study shows that the results obtained when calculating %BF from skinfold measurements in adolescent girls with type 1 diabetes need to be viewed with caution. There could, for example, be a risk of misinterpreting the relationship between body fatness and cardiovascular risk factors when using the equations assessed in this study.

New prediction equations need to be developed to improve the accuracy of estimating body fatness from skinfolds in adolescent girls with type 1 diabetes. The prediction equation developed in this study was a good match to %BF from DXA and is the first equation derived from a paediatric population with type 1 diabetes. The weakness of this model was the low number subjects we included and the lack of external validation of the equation developed as part of this study. For that reason, the equation needs to be validated in another larger population of girls with diabetes.

## Conclusion

Using skinfold thickness equations to assess body composition in adolescent girls with type 1 diabetes showed low levels of accuracy in comparison with DXA measurements as criterion method. Our observations emphasise the need for specific skinfold equations for girls with type 1 diabetes derived from a population with an appropriate range of fatness.

## Funding

This study received financial support from the Research Committee of Örebro County Council and the Swedish Child Diabetes Foundation (Barndiabetes fonden). UE was partly funded by the MRC Epidemiology Unit, University of Cambridge, Cambridge, UK (Grant MC_UU_12015/3).

## Conflicts of interest

The authors have no conflicts of interest to declare.

## References

[1] Mortensen HB , Robertson KJ , Aanstoot HJ , Danne T , Holl RW , Hougaard P , et al. Insulin management and metabolic control of type 1 diabetes mellitus in childhood and adolescence in 18 countries. Hvidore Study Group on Childhood Diabetes. Diabet Med 1998; 15: 752–9. 9737804 10.1002/(SICI)1096-9136(199809)15:9<752::AID-DIA678>3.0.CO;2-W

[2] Tylleskär K , Tuvemo T , Gustafsson J . Diabetes control deteriorates in girls at cessation of growth: relationship with body mass index. Diabet Med 2001; 18: 811–5. 11678971 10.1046/j.1464-5491.2001.00587.x

[3] Maffeis C , Morandi A , Ventura E , Sabbion A , Contreas G , Tomasselli F , et al. Diet, physical, and biochemical characteristics of children and adolescents with type 1 diabetes: relationship between dietary fat and glucose control. Pediatr Diabetes 2012; 13: 137–46. 21672107 10.1111/j.1399-5448.2011.00781.x

[4] Ahmed ML , Ong KK , Watts AP , Morrell DJ , Preece MA , Dunger DB . Elevated leptin levels are associated with excess gains in fat mass in girls, but not boys, with type 1 diabetes: longitudinal study during adolescence. J Clin Endocrinol Metab 2001; 86: 1188–93. 11238507 10.1210/jcem.86.3.7320

[5] Scottish Study Group for the Care of the Young Diabetic . Factors influencing glycemic control in young people with type 1 diabetes in Scotland: a population‐based study (DIABAUD2). Diabetes Care 2001; 24: 239–44. 11213872 10.2337/diacare.24.2.239

[6] Svensson M , Engström I , Åman J . Higher drive for thinness in adolescent males with insulin‐dependent diabetes mellitus compared with healthy controls. Acta Paediatr 2003; 92: 114–7. 12650311 10.1111/j.1651-2227.2003.tb00480.x

[7] Ingberg CM , Sarnblad S , Palmer M , Schvarcz E , Berne C , Aman J . Body composition in adolescent girls with type 1 diabetes. Diabet Med 2003; 20: 1005–11. 14632701 10.1046/j.1464-5491.2003.01055.x

[8] Riihimaa PH , Knip M , Ruokonen A , Tapanainen P . Lack of physiological suppression of circulating IGFBP‐1 in puberty in patients with insulin‐dependent diabetes mellitus. Eur J Endocrinol 2002; 147: 235–41. 12153746 10.1530/eje.0.1470235

[9] Lukacs A , Mayer K , Juhasz E , Varga B , Fodor B , Barkai L . Reduced physical fitness in children and adolescents with type 1 diabetes. Pediatr Diabetes 2012; 13: 432–7. 22353226 10.1111/j.1399-5448.2012.00848.x

[10] Slaughter MH , Lohman TG , Boileau RA , Horswill CA , Stillman RJ , Van Loan MD , et al. Skinfold equations for estimation of body fatness in children and youth. Hum Biol 1988; 60: 709–23. 3224965

[11] Durnin JV , Rahaman MM . The assessment of the amount of fat in the human body from measurements of skinfold thickness. Br J Nutr 1967; 21: 681–9. 6052883 10.1079/bjn19670070

[12] Deurenberg P , Pieters JJ , Hautvast JG . The assessment of the body fat percentage by skinfold thickness measurements in childhood and young adolescence. Br J Nutr 1990; 63: 293–303. 2334665 10.1079/bjn19900116

[13] Sloan AW , Burt JJ , Blyth CS . Estimation of body fat in young women. J Appl Physiol 1962; 17: 967–70. 13989261 10.1152/jappl.1962.17.6.967

[14] Thorland WG , Johnson GO , Tharp GD , Housh TJ , Cisar CJ . Estimation of body density in adolescent athletes. Hum Biol 1984; 56: 439–48. 6489989

[15] Parizkova J . Total body fat and skinfold thickness in children. Metabolism 1961; 10: 794–807. 14483890

[16] Sarnblad S , Ekelund U , Aman J . Physical activity and energy intake in adolescent girls with Type 1 diabetes. Diabet Med 2005; 22: 893–9. 15975105 10.1111/j.1464-5491.2005.01544.x

[17] Lohman TG , Roche AF , Martorell R . Anthropometric standardization reference manual, Abridged ed. Champaign Ill: Human Kinetics Books, 1991.

[18] Jeppsson JO , Jerntorp P , Sundkvist G , Englund H , Nylund V . Measurement of hemoglobin A1c by a new liquid‐chromatographic assay: methodology, clinical utility, and relation to glucose tolerance evaluated. Clin Chem 1986; 32: 1867–72. 3757206

[19] *equalis.se* Availabel from http://www.equalis.se: Equalis AB, [cited 2015 14 July].

[20] Bland JM , Altman DG . Statistical methods for assessing agreement between two methods of clinical measurement. Lancet 1986; 1: 307–10. 2868172

[21] Ingberg CM , Palmer M , Aman J , Arvidsson B , Schvarcz E , Berne C . Body composition and bone mineral density in long‐standing type 1 diabetes. J Intern Med 2004; 255: 392–8. 14871464 10.1046/j.1365-2796.2003.01283.x

[22] Tillmann V , Adojaan B , Shor R , Price DA , Tuvemo T . Physical development in Estonian children with type 1 diabetes. Diabet Med 1996; 13: 97–101. 8741820 10.1002/(SICI)1096-9136(199601)13:1<97::AID-DIA20>3.0.CO;2-L

[23] Rosenbloom AL , Silverstein JH . Connective tissue and joint disease in diabetes mellitus. Endocrinol Metab Clin North Am 1996; 25: 473–83. 8799711 10.1016/s0889-8529(05)70335-2

[24] Monnier VM , Bautista O , Kenny D , Sell DR , Fogarty J , Dahms W , et al. Skin collagen glycation, glycoxidation, and crosslinking are lower in subjects with long‐term intensive versus conventional therapy of type 1 diabetes: relevance of glycated collagen products versus HbA1c as markers of diabetic complications. DCCT Skin Collagen Ancillary Study Group. Diabetes Control and Complications Trial. Diabetes 1999; 48: 870–80. 10102706 10.2337/diabetes.48.4.870 PMC2862597

[25] Wong WW , Stuff JE , Butte NF , Smith EO , Ellis KJ . Estimating body fat in African American and white adolescent girls: a comparison of skinfold‐thickness equations with a 4‐compartment criterion model. Am J Clin Nutr 2000; 72: 348–54. 10919926 10.1093/ajcn/72.2.348

[26] Rodriguez G , Moreno LA , Blay MG , Blay VA , Fleta J , Sarria A , et al. Body fat measurement in adolescents: comparison of skinfold thickness equations with dual‐energy X‐ray absorptiometry. Eur J Clin Nutr 2005; 59: 1158–66. 16047030 10.1038/sj.ejcn.1602226

[27] Reilly JJ , Wilson J , Durnin JV . Determination of body composition from skinfold thickness: a validation study. Arch Dis Child 1995; 73: 305–10. 7492193 10.1136/adc.73.4.305 PMC1511327

